# A Systematic Comparison of Motion Artifact Correction Techniques for Functional Near-Infrared Spectroscopy

**DOI:** 10.3389/fnins.2012.00147

**Published:** 2012-10-11

**Authors:** Robert J. Cooper, Juliette Selb, Louis Gagnon, Dorte Phillip, Henrik W. Schytz, Helle K. Iversen, Messoud Ashina, David A. Boas

**Affiliations:** ^1^Department of Radiology, Athinoula A. Martinos Center for Biomedical Imaging, Massachusetts General Hospital, Harvard Medical SchoolCharlestown, MA, USA; ^2^Harvard-MIT Division of Health Sciences and TechnologyCambridge, MA, USA; ^3^Department of Electrical Engineering and Computer Science, Massachusetts Institute of TechnologyCambridge, MA, USA; ^4^Danish Headache Center, Department of Neurology, Glostrup Hospital, Faculty of Health Sciences, University of CopenhagenCopenhagen, Denmark; ^5^Stroke Unit, Department of Neurology, Glostrup Hospital, Faculty of Health Sciences, University of CopenhagenCopenhagen, Denmark

**Keywords:** near-infrared spectroscopy, functional near-infrared spectroscopy, NIRS, motion artifact, hemodynamic response

## Abstract

Near-infrared spectroscopy (NIRS) is susceptible to signal artifacts caused by relative motion between NIRS optical fibers and the scalp. These artifacts can be very damaging to the utility of functional NIRS, particularly in challenging subject groups where motion can be unavoidable. A number of approaches to the removal of motion artifacts from NIRS data have been suggested. In this paper we systematically compare the utility of a variety of published NIRS motion correction techniques using a simulated functional activation signal added to 20 real NIRS datasets which contain motion artifacts. Principle component analysis, spline interpolation, wavelet analysis, and Kalman filtering approaches are compared to one another and to standard approaches using the accuracy of the recovered, simulated hemodynamic response function (HRF). Each of the four motion correction techniques we tested yields a significant reduction in the mean-squared error (MSE) and significant increase in the contrast-to-noise ratio (CNR) of the recovered HRF when compared to no correction and compared to a process of rejecting motion-contaminated trials. Spline interpolation produces the largest average reduction in MSE (55%) while wavelet analysis produces the highest average increase in CNR (39%). On the basis of this analysis, we recommend the routine application of motion correction techniques (particularly spline interpolation or wavelet analysis) to minimize the impact of motion artifacts on functional NIRS data.

## Introduction

Near-infrared spectroscopy (NIRS) is a burgeoning neuro-investigatory technique that uses measures of the intensity of diffusely scattered near-infrared light to calculate changes in oxyhemoglobin concentration (HbO) and deoxy-hemoglobin concentration (HbR) in cortical tissues (Obrig et al., 2000; Obrig and Villringer, 2003; Lloyd-Fox et al., 2010). The majority of NIRS studies are performed using a back-reflection geometry, with near-infrared light carried to and from the head via optical fibers (Obrig et al., 2000). Although the methods of coupling optical fibers to the scalp have improved since the inception of NIRS technologies (Lloyd-Fox et al., 2010), movement of the head of the subject will invariably cause motion between each optical fiber and the scalp. This relative motion will cause a rapid shift in the optical coupling between the fiber and the scalp, which typically results in a period of high-frequency noise in the recorded NIRS data. Even after the motion has subsided, the optical coupling is often irrevocably altered, causing a shift in the baseline measurements of HbO and HbR. Because the magnitude of this motion-induced noise is typically far greater than the changes associated with tissue hemodynamics, any changes in hemoglobin concentrations in the cerebral cortex which coincide with motion artifacts are usually so heavily contaminated that they are indiscernible.

Motion artifacts in NIRS data are usually relatively easy to identify using a combination of observation of the subject during NIRS recording and visual inspection of the resulting data. There are also a number of approaches to the automated detection of motion artifacts, using algorithms which identify changes in NIRS signals which are of a scale, rate or nature that are unlikely to be physiological (Cui et al., 2010; Scholkmann et al., 2010; Cooper et al., 2011). However, once motion artifacts have been identified, there is no well-established approach to their removal or correction. Instead, the standard approach to motion artifacts in functional NIRS data is to either attempt to insure that there are enough stimulus trials to minimize the average impact of the artifacts or simply to remove any stimulus trials that coincide with identified artifacts. Both of these approaches have limitations. Subject groups that tend to exhibit extensive motion are often also the groups for whom the duration of NIRS recording is limited. In such cases the number of stimulus trials is also limited. This can make it impossible for motion artifacts to be removed by a process of averaging, while trial rejection can reduce the effective number of stimulus trials even further, reducing the contrast-to-noise ratio (CNR) of the estimated hemodynamic response function (HRF).

Motion artifacts are typically observable in multiple NIRS channels and have a scale and frequency composition that are distinct from the background NIRS signal. For these reasons, a variety of signal processing approaches have emerged that attempt to reduce or remove motion artifacts from functional NIRS data, in order that the corrected data can contribute to the estimated HRF. In the context of NIRS experimental design, these motion correction methods fall in to two distinct categories: those which require some additional input beyond the standard NIRS dataset (and therefore require an alteration of experimental design) and those that do not.

The first category encompasses a variety of approaches which require an input signal that is highly correlated with the NIRS motion artifacts but not with the NIRS functional response. Such an input signal can be obtained via an external measure of motion [such as an accelerometer (Blasi et al., 2010; Izzetoglu et al., 2010; Virtanen et al., 2011)] but will more commonly be a reference NIRS signal (Robertson et al., 2010). A reference NIRS signal can be acquired simultaneously with the standard functional NIRS experiment using an additional NIRS channel which, (due to its location or source-detector separation) is not sensitive to the functional response of interest. This additional input signal can then be used to cancel motion artifacts from NIRS channels via linear regression, a variety of adaptive filtering approaches and Wiener filtering (Izzetoglu et al., 2005; Zhang et al., 2009; Robertson et al., 2010).

Although these approaches show great promise, we wish to assess the utility of motion correction techniques which fall into the second category, i.e., approaches which can be applied to standard functional NIRS datasets without alteration of the experimental paradigm. Such post-processing techniques typically take advantage of the distinct amplitude and frequency characteristics of motion artifacts in order to remove them. Perhaps the most common example is an application of principle component analysis (PCA). PCA is a process of orthogonal linear transformation which can be used to isolate components corresponding to motion artifact with minimal impact on the physiological components present in the NIRS data. This approach was described by Zhang et al. (2005) and similar approaches have been applied in a number of functional NIRS studies (Wilcox et al., 2008, 2010).

In recent years, several other approaches to motion artifact correction have been put forward which do not require additional input measurements. These include the spline interpolation approach suggested by Scholkmann et al. (2010), in which periods of motion are modeled using a cubic spline interpolation and this model is then subtracted from the original signal. Signal filtering based on a discrete wavelet transform has also been applied to the removal of motion artifacts from NIRS data (Molavi and Dumont, 2012). Because motion tends to result in abrupt changes in the frequency content of the NIRS signal, wavelet transformation can be very effective in isolating motion artifacts. The coefficients which correspond to motion artifacts in the wavelet domain can then be removed before the NIRS data is reconstructed. Lastly, discrete Kalman filtering has been applied to the removal of motion artifacts from NIRS data (Izzetoglu et al., 2010). A Kalman filter is a recursive, state-space based approach to the recovery of signals that are contaminated by noise and (assuming the NIRS time-course contains some periods of data that are motion-free) can be applied without the need for additional inputs.

Despite the prevalence and variety of motion artifact correction techniques, there is little evidence to suggest that the application of any of these approaches to functional NIRS actually improves the accuracy or CNR of the recovered HRF. In previous reports, the metrics applied to test the utility of a given motion correction technique have been varied but insufficient. In cases where real motion artifact contaminated NIRS data has been used, the utility of a correction technique has been quantified using some measure of improvement in signal-to-noise ratio of the corrected NIRS time-course (Izzetoglu et al., 2010; Scholkmann et al., 2010; Molavi and Dumont, 2012). In some cases, motion artifacts themselves have been simulated and added to real NIRS data and subsequently corrected, which allows the mean-squared error (MSE) and Pearson’s correlation coefficient (*R*^2^) between the original and corrected signals to be calculated (Scholkmann et al., 2010). However, motion artifacts are complex and variable, and difficult to accurately simulate, particularly across multiple channels. Scholkmann et al. (2010) did apply their spline motion correction technique to real NIRS motion artifacts which occurred during a functional activation experiment, but it was not possible to meaningfully quantify the improvement because the true hemodynamic response was unknown.

In this paper we present a systematic comparison of four recently promoted motion correction algorithms by applying them to real motion artifact contaminated NIRS data. By adding a number of synthetic HRFs to each dataset and then attempting to recover the average HRF, we are able to simulate a functional NIRS study and assess each motion correction technique in terms of the improvement in the accuracy of the recovered HRF. The accuracy of this recovery can then be compared to that achieved without motion correction and by simply removing motion-contaminated trials entirely.

## Materials and Methods

### NIRS data

Twenty NIRS datasets were selected from a clinical study of adult stroke patients at Glostrup Hospital in Copenhagen, Denmark. Each dataset consists of 10 min of resting-state NIRS recording using eight dual-wavelength, 3 cm separation channels positioned across the forehead and fronto-central areas of the head. These datasets were obtained using a TechEn Inc. CW6 system (Medford, MA, USA) which employs frequency multiplexed sources at 690 and 830 nm with a sample rate of 25 Hz. This system is described in detail by Franceschini et al. (2003).

The clinical condition of the patients meant that motion artifacts were common, despite the patients being asked to lie still during recording. These 20 datasets were chosen on the basis that they all contained motion artifacts as determined on visual inspection by two investigators (Robert J. Cooper, Juliette Selb). The motion artifact identification algorithm *hmrMotionArtifact* from the HOMER2 NIRS processing package^1^ (Huppert et al., 2009) was applied to determine periods of motion artifact in each of the 20 NIRS datasets. This algorithm provides reliable identification of motion artifacts based on changes in signal amplitude and/or standard deviation and is similar to the approach described by Scholkmann et al. (2010) and Cooper et al. (2011). If the standard deviation increases by a factor exceeding *SDThresh*, or the peak-to-peak amplitude exceeds *AMPThresh*, both within a window of length *tMotion*, then data from the beginning of that window to *tMask* seconds later is defined as motion. Values of *SDThresh* = 20, *AMPThresh* = 0.5, *tMotion* = 0.5 s and *tMask* = 2 s are effective in most cases. However, as we did not wish to test the validity of this detection approach here and simply needed to identify motion as accurately as possible, *SDThresh* was fixed individually for each dataset (at values between 12 and 20) in order to maximize the accuracy of motion identification as determined by visual inspection. (Note that the output of this algorithm is not channel-specific, i.e., signal changes which are determined to be artifact in one channel are marked as motion in all channels; this relies on the reasonable assumption that motion artifacts affect multiple channels).

Across the 20 datasets, an average of 10.6% of each NIRS dataset was identified as motion, ranging from 1.9 to 28.6%. The average duration of a given period of artifact was 4.2 s (though note that *tMask* = 2 s determines the minimum length of each motion artifact). This suggests that the majority of artifacts are brief, and likely take the form of transient spikes. This is consistent with our experimental experience.

### Motion correction techniques

#### Principle component analysis

The application of PCA transforms an *N*-measurement NIRS dataset into *N* linearly uncorrelated components, ordered by their contribution to the variance of the data. The magnitude of motion artifacts is typically much larger than the background NIRS signal. Motion artifacts are also typically present on multiple NIRS channels. The application of PCA to the filtering of NIRS data makes the assumption that motion artifacts provide the dominant contribution to the variance of the NIRS data, and that therefore the first *r* principle components will account purely for motion artifact. These *r* components can then be removed before the data is analyzed further. For a NIRS dataset Y, of dimensions time × channel, the PCA motion correction consists of first performing singular value decomposition on the spatial correlation matrix *Y^T^Y* such that:

$$Y^{T}Y=U\Omega U^{T}$$

where ***U*** is the orthogonal matrix of spatial eigenvectors and **Ω** is a diagonal matrix of the associated eigenvalues, both of dimensions *N* × *N* (Zhang et al., 2005). The first ***r*** eigenvectors can then be removed from the data, such that:

$$Y_{corrected}=Y\left(I-UA_{r}U^{T}\right)$$

where A_r_ is a *N* × *N* matrix with the first *r* diagonal elements equal to 1, and all other elements equal to zero. The performance of PCA in removing motion artifacts from NIRS data is heavily dependent on two factors: the number of NIRS measurements in a dataset [as this defines the number of components (*N*)] and the number of components that are removed (*r*). Note that the number of NIRS measurements is twice the number of channels as we define them: each channel consists of measurements at two different wavelengths, in our case therefore, *N* = 16. The optimum value of the parameter *r* will clearly depend on the total number of components (*N*) and on how many of those components constitute artifact, which will change from dataset to dataset.

A simple way to approach the selection of *r* is to normalize the diagonal elements of the matrix **Ω** by their sum to produce the vector $\tilde{\Omega }$. The *n*th element of $\tilde{\Omega }$ then provides a measure of the proportion of the NIRS data variance that is accounted for by the *n*th component. By fixing the proportion of data variance that is to be removed (referred to from herein as σ*_PCA*), the number of components to remove (*r*) can be defined by finding the lowest value of *r* for which:

$$\overset{r}{\underset{n=1}{\sum }}{\tilde{\Omega }}_{n}\geq \sigma \text{\_}PCA.$$

#### Spline interpolation

The spline interpolation approach applied here is that described by Scholkmann et al. (2010). Once periods of motion artifact have been defined (performed here using *hmrMotionArtifact* from HOMER2) each of those periods is modeled one by one, throughout each NIRS time-course using the MATLAB^™^ cubic spline interpolation function *csaps*. Each period of modeled data is then subtracted from that period of the original data. In order to produce a continuous signal all the data points after the start of the corrected motion segment after shifted by a constant value. This value is defined as the difference between the mean of the signal at the start of the corrected motion period and the mean of the signal prior to the corrected motion period. The durations over which these means are calculated must be variable, as the length of motion artifacts and the length of the data prior to each motion artifact is also variable. These durations were defined using the framework set out in Scholkmann et al. (2010).

The spline interpolation depends on the interpolation parameter *p_Spline*. This parameter is defined in MATLAB^™^ such that if *p_Spline* = 0, the motion artifact is modeled with a least-squares straight-line fit. If *p_Spline* = 1, the motion artifact is modeled via a natural cubic spline interpolation. Therefore, performing motion correction with *p_Spline* = 0 will remove only a straight-line fit from the artifact while a *p_Spline* = 1 model will approximate the artifact very accurately and on subtraction leave a near-constant value. Scholkmann et al. (2010) define their parameter as (*1−p_Spline*) and suggest that a value corresponding to *p_Spline* = 0.99 is reliably effective in the removal of motion artifacts.

#### Wavelet analysis

We employed the discrete wavelet analysis and filtering approach described by Molavi and Dumont (2012). While a detailed description of wavelet decomposition is not provided here (see Strang and Nguyen, 1996; Molavi and Dumont, 2012) in summary each NIRS data time-course, *y*(*t*), is transformed into the wavelet domain using the general discrete wavelet transformation:

$$y\left(t\right)=\underset{k}{\sum }v_{j_{0}k}\phi_{j_{0}k}\left(t\right)+\overset{\infty }{\underset{j=j_{0}}{\sum }}\underset{k}{\sum }\omega_{jk}\psi_{jk}\left(t\right).$$

Here, ϕ*_jk_*(*t*) and ψ*_jk_*(*t*) are the scaling and wavelet functions and $v_{j_{0}k}$ and *ω_jk_* are the approximation and detail coefficients respectively. Indices *j* and *k* are the wavelet dilation and translation parameters with *j*_0_ the coarsest decomposition. After decomposition, the model assumes that the NIRS signal consists of meaningful physiological signals *f*(*t*) plus artifact terms *e*(*t*) such that *y*(*t*) = *f*(*t*) + *e*(*t*) and assumes the wavelet coefficients (ω*_jk_*) exhibit a Gaussian probability distribution. Because the physiological hemodynamic signal is generally smooth compared to motion artifact, the distribution of the wavelet coefficients that represent the physiological components of the NIRS signal will be centered around zero and have a low variance, whereas those corresponding to motion artifact will appear as outliers in the probability distribution. In order to remove these motion artifact coefficients before performing the inverse wavelet transformation to reconstruct the NIRS time-course, we apply a probability threshold to remove outlying wavelet coefficients. For a given wavelet coefficient ω*_jk_*, if the probability of observing values greater than ω*_jk_* is less than a threshold value α*_Wav*, then we assume that the coefficient corresponds to motion artifact and it is set to zero. The probability threshold α*_Wav* is a tunable parameter and a correct choice of its value is essential to the performance of wavelet analysis in the removal of motion artifacts from NIRS data. This analysis employed the Wavelab 850 toolbox for MATLAB^™^^2^.

#### Discrete Kalman filtering

The approach described by Izzetoglu et al. (2010) was the basis for our Kalman filtering of NIRS motion artifacts. Kalman filtering is a state-based recursive technique that acts on noisy data to provide a statistically optimal estimate of the underlying signal and is essentially a two-step process. The first is to use prior knowledge of a state to make a prediction of a future state and its uncertainty. This prediction step models the state *x* at time = *k*, based on the state at time = *k−1* such that:

$$x_{k}=Ax_{k-1}+\gamma_{k}$$

where *A* is the transition model (which uses knowledge of the prior state to predict the current state) and γ*_k_* is the system noise. This prediction is then compared to the actual measured state at time = *k*, *z_k_*, which is modeled as a function of the true state *x*:

$$Z_{k}=Hx_{k}+\delta_{k}$$

where *H* is the observation model that maps the true state-space into the measurement space and δ*_k_* is the measurement noise. The second step is to use the prediction *x_k_*, the measurement *z_k_*, and their error covariances to produce an updated estimate of the true state, which is determined by the Kalman gain. The resulting optimal estimator is then fed back in to the prediction step as the prior state and the process is repeated to find the optimal estimator of the (*k*+1)th state. For more details see Grewal and Andrews (2003) and Izzetoglu et al. (2010).

For the application of the Kalman filter to the reduction of NIRS motion artifacts, the translation model was an auto-regressive model based on the Yule–Walker method, with a model order *M* = 4. This was the highest model order that remained stable on application to our data, and is also that recommended by Izzetoglu et al. (2010). The *M* Yule–Walker coefficients were calculated on a measurement-by-measurement basis by taking the correlation between the longest period of motion-free data and itself, offset by 1 to *M* data points. Prior to Kalman filtering, the NIRS data was down-sampled to 1 Hz. The system and measurement noise were taken to be the variance of the motion-free NIRS data and the variance of the entire time-course respectively for each NIRS measurement. As throughout this paper, periods of motion were determined by the algorithm *hmrMotionArtifact*.

**Figure 1:**
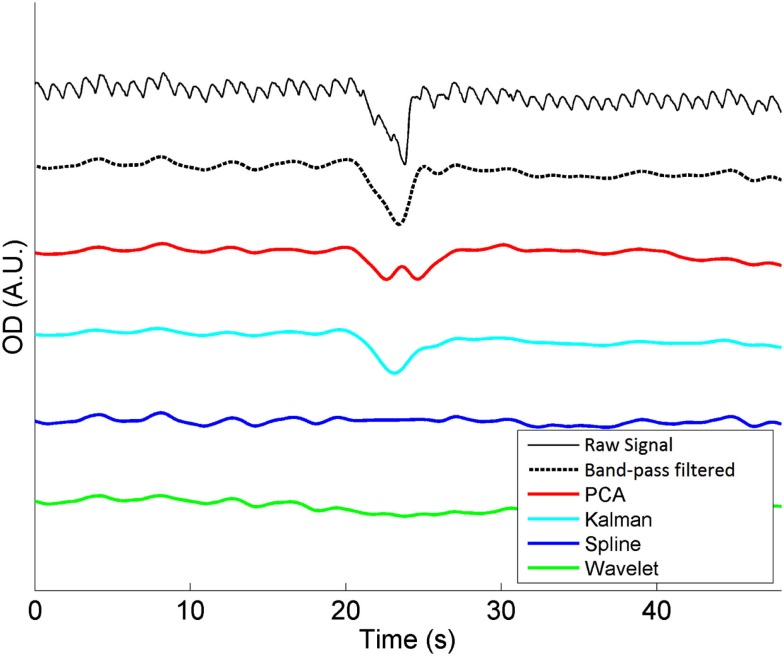
**A single motion artifact from one of our 20 NIRS datasets and the effect of band-pass filtering (between 0.01 and 0.5 Hz), PCA, spline interpolation, wavelet analysis, and Kalman filtering on that artifact**. Note that the same band-pass filter is also applied after each motion correction technique.

Figure 1 shows an example of a single motion artifact in a period of NIRS data, and the results of the correction of that motion artifact using a band-pass filtering, PCA, spline interpolation, wavelet analysis, and Kalman filtering (all also in combination with the same band-pass filter, applied after the motion correction).

### Simulated functional NIRS study, HRFs and data processing

The simulation paradigm, performed using the MATLAB^™^ computing environment, proceeded as follows: First, all raw optical intensity measurements for each of the 20 datasets were converted into changes in optical density (OD). A timing vector, which provides 25 trial onset positions spaced randomly throughout the 10 min dataset but with a minimum separation of 20 s was then constructed. This vector mimics the experimental design of a typical functional NIRS experiment, with each onset position corresponding to the beginning of a functional task. A simulated HRF was then designed that consisted of a gamma function with a time-to-peak of 7 s, a duration of 20 s and an amplitude defined so as to produce a 15 μM increase in HbO concentration and a 5 μM decrease in HbR concentration [these figures include a partial volume correction factor of 50 (Strangman et al., 2003)]. Such amplitudes are consistent with those observed in real NIRS studies and have also been used in previous functional NIRS simulations (Gagnon et al., 2011, 2012). These amplitudes are approximately equivalent to an intensity change of 0.9% from baseline for the 690 nm channels and 2% from baseline for the 830 nm channels.

Three of the eight NIRS channels from each of the 20 NIRS datasets were randomly selected and the simulated HRF was added to the OD time-course of these channels at the 25 onset positions defined by the timing vector. Lastly, the automatic motion detection algorithm *hmrMotionArtifact* was applied to each NIRS dataset to define periods of motion *after* the simulated HRFs had been added to the data.

Once the simulated functional dataset had been completed, it was then passed through six different processing streams. The first was the recovery of the HRF without motion correction or the rejection of trials. The data was band-pass filtered (a third order Butterworth filter between 0.01 and 0.5 Hz) to remove low-frequency drift and cardiac oscillations before being converted into HbO and HbR using the modified Beer–Lambert law (Obrig et al., 2000). The periods of data around each of the 25 stimulus trials were then block-averaged to produce the mean HRF. The second processing stream was a standard trial rejection NIRS processing approach. After band-pass filtering the output of *hmrMotionArtifact* (calculated previously) was applied such that if a stimulus trial coincided with a defined period of motion, then that stimulus was rejected. The remaining stimuli periods were then block-averaged in order to recover the mean HRF.

The first step of the remaining four processing streams consisted of the implementation of each of the four motion correction techniques: PCA, spline, wavelet, and Kalman respectively. The resulting corrected data was then subjected to the same band-pass filter as the standard processing approaches, followed by conversion to HbO and HbR. All stimulation trials, irrelevant of how effective the motion correction process may have been, were then included in the block-average calculation of the mean HRF.

This entire process was repeated five times for each dataset using a different random selection of channels and a different stimulus timing vector in order to improve the robustness of the results.

### Metrics for comparison

The result of the simulation process described above was 300 HRFs (20 datasets × 3 channels × 5 repetitions) for each of the six processing streams (no correction, trial rejection, PCA, spline, wavelet, Kalman). Three metrics were then calculated for each processing stream. These consisted of the MSE between each recovered HRF and the true (simulated) HRF, the Pearson’s correlation coefficient (*R*^2^) between the recovered and true HRF and finally the CNR of the recovered HRF. The CNR was calculated by taking the mean of the 2 s of recovered HRF data centered at the true HRF peak time (7 s after onset) and dividing by the standard deviation of the 5 s of data prior to onset. These three metrics allow a direct comparison of the accuracy of the HRFs recovered via standard NIRS processing approaches and via each motion correction method. All metrics are calculated on the basis of the recovered HbO signal only for computational simplicity.

### Sensitivity analyses

Several of the motion correction techniques applied here require the selection of an input parameter that can significantly affect the performance of the technique. For PCA, spline, and wavelet approaches, these parameters are the percentage variance to remove (σ*_PCA*), the interpolation parameter (*p_Spline*) and the probability threshold (α*_Wav*) respectively and are described above. In order to optimize the performance of each of these methods, it was first necessary to perform a sensitivity analysis for each of these three techniques. The simulation described above was repeated, in full, for a variety of values for each input parameter. Guided by the values of these parameters applied in previous publications, σ*_PCA* was varied from 50 to 99.9%, *p_Spline* was varied from 0 to 1, and α*_Wav* was varied from 0.01 to 0.8.

## Results

### Trial rejection vs. no correction

**Figure 2:**
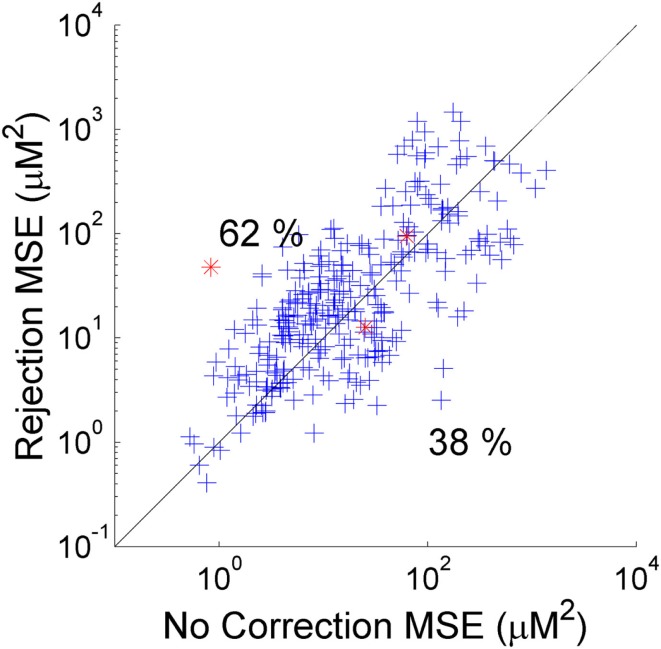
**The MSE between the recovered and true HRF for 300 simulations using trial rejection (*y*-axis) and no correction (*x*-axis)**. Trial rejection produces an increase in MSE in 62% of cases, but there is no statistically significant difference between the MSE achieved by trial rejection and that of no correction. The red asterisks indicate the simulations corresponding to the HRFs shown in Figure 3.

As described above, the two standard approaches to motion artifacts in functional NIRS data are to perform no correction, or to simply reject those trials which coincide with motion and not include them in the calculation of the mean HRF. Which of these two approaches produces the best recovered HRF will clearly depend on the nature and extent of the motion artifacts in each dataset and the number of stimulus trials. In our simulation the number of trials rejected (out of a possible 25) varied from 7 to 23 and averaged 13.9 across the 20 NIRS datasets and 5 repetitions. Figure 2 provides a scatter plot of the MSE between the 300 recovered HRFs and the simulated HRF for trial rejection (*y*-axis) vs. no correction (*x*-axis). In 62% of cases, trial rejection actually increases the MSE. However in this simulation the difference between the MSE for the trial rejection and no correction approaches is not significant in a two-tailed paired *t*-test (*p* = 0.18).

### Sensitivity analyses

The sensitivity analyses for input parameters σ*_PCA*, *p_Spline*, and α*_Wav* allowed us to determine the values that minimized MSE and maximized *R*^2^ and CNR. In all three cases, a single value of each parameter produced the best result for all three metrics. These were σ*_PCA* = 97%, *p_Spline* = 0.99, and α*_Wav* = 0.1. The optimized parameter value for wavelet analysis, α*_Wav* = 0.1, is close to the figure of 0.15 calculated by Molavi and Dumont (2012). The optimized value of *p_Spline* is equal to that suggested by Scholkmann et al. (2010) in their own analysis. These three optimized parameter values were employed in all remaining analyses.

### PCA, spline, wavelet, and Kalman vs. no correction

**Figure 3:**
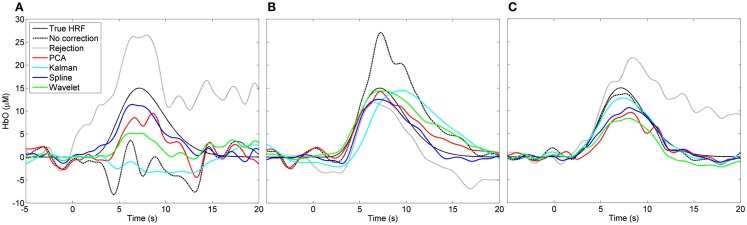
**Examples of recovered hemodynamic response functions**. **(A)** Is a case where the initial (“no correction”) HRF is very poor and PCA, spline, and wavelet approaches can be seen to improve it. **(B)** Is a case where the initial HRF is reasonably accurate but likely contaminated by motion. Here the HRF is improved by trial rejection and all four motion correction techniques. **(C)** Shows a case where the initial HRF is very accurate. Trial rejection is detrimental to the HRF as are PCA, spline, and wavelet corrections.

**Figure 4:**
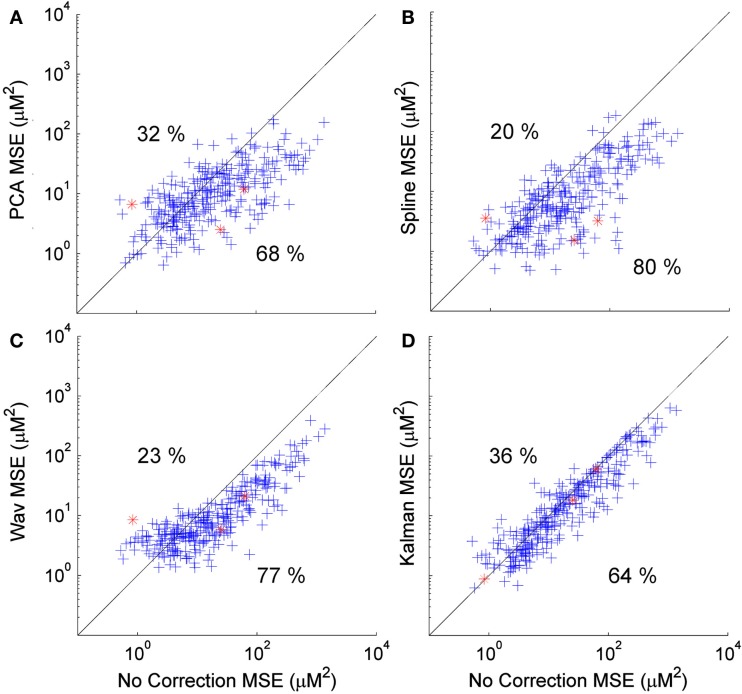
**Scatter plots showing the MSE of each HRF recovered via PCA (A), spline (B), wavelet (C), and Kalman (D) techniques, plotted against that achieved by no correction**. A clear trend is apparent for wavelet analysis (and to a lesser extent PCA) that indicates a tendency for the MSE to be increased in cases where the initial (no correction) value was low, while a decrease in MSE is reliably achieved if the initial MSE is high.

**Figure 5:**
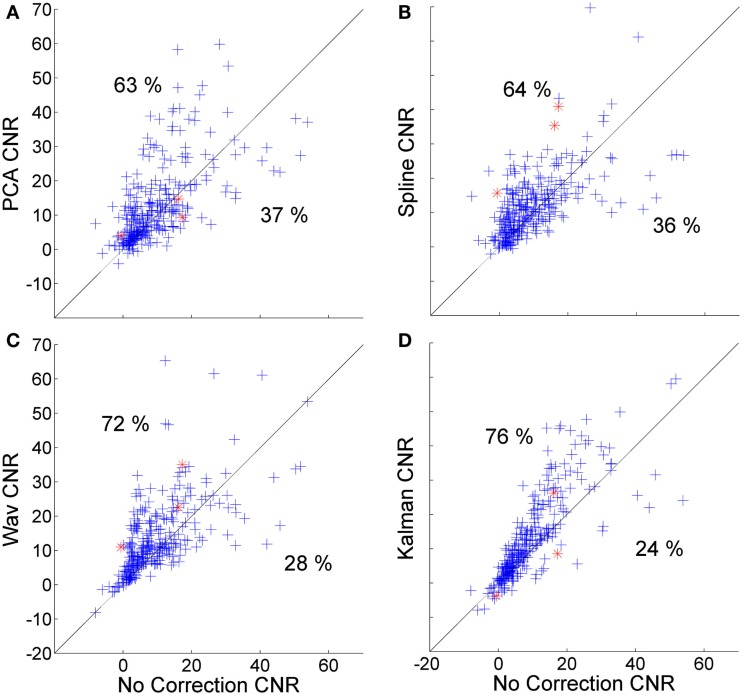
**Scatter plots showing the CNR of each HRF recovered via PCA (A), spline (B), wavelet (C), and Kalman (D) techniques, plotted against that achieved by no correction**.

Figure 3 shows the result of three individual HRF simulations. The true HRF and the HRFs recovered by all six processing streams are shown for a case where the uncorrected HRF appears very poor (Figure 3A), a case where the uncorrected HRF appears reasonable (Figure 3B), and a case where the uncorrected HRF is very close to the true HRF (Figure 3C). In Figures 2, 4, and 5 these three simulation examples are marked in red for comparison.

Figure 4 shows scatter plots of the MSE of the 300 HRFs recovered via PCA (Figure 4A), spline (Figure 4B), wavelet (Figure 4C), and Kalman (Figure 4D), plotted against those achieved with no correction. In all cases, the MSE decreases significantly (*p* < 0.01, one-tailed paired *t*-test). Note that the decrease is also significant for all four techniques when compared to the trial rejection approach. All four techniques produce an increase in MSE in a subset of cases (32, 20, 23, and 36% for PCA, spline, wavelet, and Kalman respectively). All four techniques also show a trend which indicates that motion correction is much more likely to have a detrimental effect if the initial (no correction) HRF has a low MSE, i.e., if the HRF is already very accurate. This is particularly clear for wavelet analysis, where an increase in MSE is common if the uncorrected value was below 10 μM^2^, while a decrease in MSE is assured if wavelet analysis is applied when the uncorrected MSE is greater than 20 μM^2^.

Figure 5 shows the corresponding scatter plots for the CNR of the 300 HRFs recovered via PCA (Figure 4A), spline (Figure 4B), wavelet (Figure 4C), and Kalman (Figure 4D), plotted against those achieved with no correction. Again, all four techniques produce a significant improvement (*p* < 0.01, one-tailed *t*-test). This is also the case when compared to the trial rejection approach. The distributions shown in Figures 5A–D indicate that the effect of motion correction in cases where the initial CNR is high is extremely variable. By contrast, cases where the initial CNR was between approximately 5 and 15 show a consistent improvement, particularly for wavelet and Kalman filtering.

### Overall simulation results

**Figure 6:**
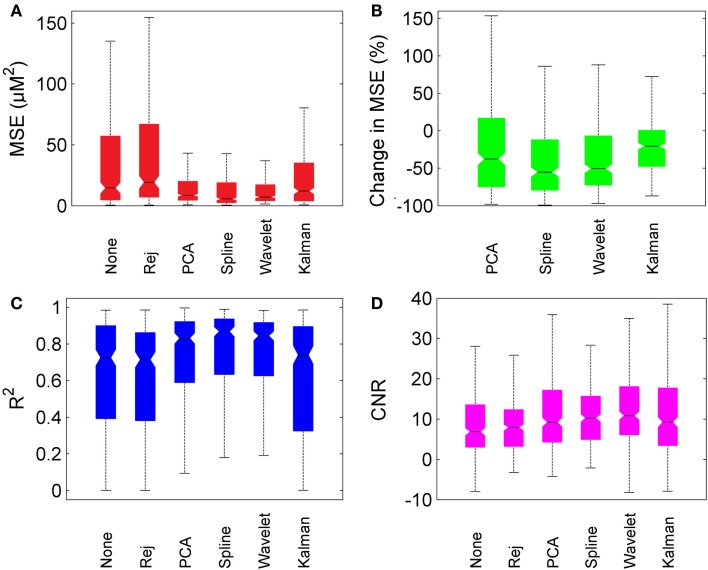
**Overall motion correction results**. Each box plot shows the median (line and notch), the 25th and 75th percentiles (the edges of each box) and the most extreme values not considered outliers (whiskers). **(A)** shows the MSE for no correction (“None”), trial rejection (“Rej”), and each of the four motion correction techniques. **(B)** shows the percentage change in MSE relative to no correction. **(C,D)** show *R*^2^ and CNR for no correction (“None”), trial rejection (“Rej”), and each of the four motion correction techniques.

Figure 6 provides box-plots of the results of each motion correction technique and allows for direct comparison. Figure 6A shows the absolute MSE for no correction, trial rejection, PCA, spline, wavelet, and Kalman techniques. Figure 6B shows the pair-wise percentage change in MSE achieved by each motion correction technique compared to that achieved by no correction. Figures 6C,D show the measurements of *R*^2^ and CNR respectively.

In general, PCA, spline, and wavelet techniques all perform well, Kalman filtering less so. PCA, spline, and wavelet approaches produce a median decrease in MSE of 38, 55, and 50% respectively, compared to no correction. Kalman filtering produces a decrease of only 21%. All four methods produce an increase in *R*^2^, but while the increase produced by PCA, spline, and wavelet approaches are highly significant (*p* < 0.01) the change due to Kalman filtering is not significant (*p* > 0.05). All four methods also produced a significant increase in CNR, with wavelet filtering yielding the highest median CNR at 10.9 compared to 6.9 for no correction. The pair-wise percentage change in CNR (not shown) was also greatest for wavelet analysis, which produced a median increase of 39%.

## Discussion and Conclusion

Although many approaches to the correction or minimization of motion artifacts in NIRS data have been put forward (Izzetoglu et al., 2005, 2010; Zhang et al., 2005; Robertson et al., 2010; Scholkmann et al., 2010; Molavi and Dumont, 2012), this is the first time that the utility of these techniques have been explicitly and systematically tested for functional NIRS studies. This is important because functional studies, in which obtaining an HRF is the experimental goal, are by far the most common application of NIRS. Furthermore, functional NIRS is becoming more and more popular, in a variety of fields, and amongst users who may not wish to investigate complex signal analysis techniques themselves. One practical goal of this study was therefore to determine the motion correction techniques that can reliably improve the recovered HRF, so that those techniques can be incorporated into the open-source NIRS processing package HOMER2^3^, and disseminated to users.

The simulations performed here are designed to closely mimic a real functional NIRS experiment, but they still have a number of limitations, and our results will not apply to all datasets and all experimental paradigms. For example, the performance of PCA is heavily dependent on the number of NIRS channels in a dataset, which we did not vary here. Another potential limitation is the choice of dataset. It is possible that the types of motion artifact, the background hemodynamic oscillations and the quality of NIRS recording are different in these 20 stroke patients than in a standard functional NIRS study. However, we believe these effects are likely to be negligible.

One surprising result is that trial rejection does not produce a significant improvement in the recovered HRF compared to no correction (Figure 2). This is almost certainly due to the range of the number of trials rejected in these datasets. The minimum number of trials rejected in our simulations was 7 out of 25, which is a relatively high proportion. It is extremely likely that trial rejection would provide a significant improvement compared to no correction if only a small number of trials were initially rejected. As the proportion of rejected trials increases, the more likely it is that including those trials will produce a better HRF than rejecting them, as motion artifacts will be minimized simply by block-averaging.

All four motion correction techniques produce an improvement in HRF recovery on average. Spline interpolation provides the highest average decrease in MSE (55%), reduces MSE in 80% of cases (Figure 4) and produces the highest average *R*^2^ (Figure 6) while wavelet analysis provides an average decrease in MSE of 50%, reduces MSE in 77% of cases and produces the highest average CNR (Figure 6). Although defining a single, best technique depends on which metrics are considered, it is clear that spline and wavelet techniques are the most successful.

The results of Figures 4–6 show that in practice there will be cases where motion correction will be detrimental. The distributions shown in Figures 4A–D suggest that this is much more likely in cases where the uncorrected HRF is already very accurate, particularly for wavelet analysis. These cases of low initial MSE are the cases where the number and/or amplitude of motion artifacts is/are small. In cases where only a small proportion of stimulus trials are affected by motion artifacts, correction approaches may not be suitable and the standard approach of trial rejection would likely be preferable. This result is clearly expected for PCA and wavelet approaches. In cases where movement is rare, the assumptions on which the PCA and wavelet approaches are based will begin to break down and both techniques will begin to remove components of the data that have a physiological origin.

The fact that wavelet analysis exhibits a clear relationship between the uncorrected MSE and the improvement it can provide may give it an advantage over spline interpolation, where such a pattern is less clear. It may be possible to define a threshold (based on the proportion of data that is affected by motion artifact or the uncorrected CNR) that will determine whether the application of wavelet analysis is suitable and therefore avoid cases where wavelet analysis will be detrimental.

Although we have attempted to apply these pre-published motion correction techniques in the manner described by their respective authors, there are a number of ways in which these techniques could potentially be improved without the need for additional external inputs or for alteration of an experimental paradigm. One obvious example is to target the PCA and Kalman approaches so as to only examine periods of data that are pre-determined as motion. Wavelet analysis takes advantage of the abnormal spatial and temporal characteristics of motion artifacts in the wavelet domain to identify and remove them. Spline interpolation requires knowledge of the location of motion artifacts throughout the dataset, and interpolation is only performed for those periods. However, PCA and Kalman filtering are applied to the entire NIRS time-course, including periods where we do not believe there is motion artifact. These algorithms could easy be altered so as to only be applied during periods of identified motion artifact. The variance of the NIRS signal that is due to motion artifact during periods identified as motion artifact will obviously be very large. Applying PCA with a high value of σ*_PCA* to only those periods may produce better results that applying PCA to the entire dataset, particularly in cases where motion is not particularly prevalent and therefore accounts for a lesser proportion of the variance of the entire dataset. Another obvious advance would be to employ trial rejection in conjunction with a given motion correction technique. It would be trivial to apply *hmrMotionArtifact* to identify motion artifacts still present in the *corrected* NIRS data and remove any stimulus trials that remain contaminated. One can also conceive of a recursive technique whereby motion correction is performed repeatedly until no motion artifacts are detected.

As described in the introduction, we set out to test only those motion correction techniques that can be applied after NIRS data acquisition, without altering the experimental design and without the need for additional external inputs. While we believe this is the most relevant approach to motion correction for the majority of NIRS applications at present, this may in fact change.

The use of short separation NIRS channels (which we would define as channels with a source-detector separation of less than 10 mm in adults) has been shown to be extremely beneficial to functional NIRS studies because they provide an explicit measure of NIRS signal components originating from superficial (i.e., non-cortical) tissues (Saager and Berger, 2008; Zhang et al., 2009; Gagnon et al., 2011, 2012; Saager et al., 2011). The use of short separation channels provides some of the advantages of diffuse optical tomography (in that the cortical signal of interest can be disentangled from the superficial signal), but without the need for a very large number of channels or a dense optical fiber array. The analysis by Robertson et al. (2010) shows that short separation channels can also be very useful in the reduction of motion artifacts using adaptive filtering techniques. We anticipate that a Kalman filtering approach, using the short separation signal as an input as performed by Gagnon et al. (2011, 2012) would also be very effective in the removal of motion artifacts.

Overall, the analysis of the impact of post-processing motion correction techniques on functional NIRS data described here allows us to conclude that motion correction techniques can and should be used to improve the accuracy of the recovered HRF in functional NIRS studies that are affected by motion artifacts, especially in cases where motion artifacts are prevalent. Our results show that, on average across 20 datasets and multiple repetitions, PCA, spline interpolation, and wavelet techniques all produced significant improvements in MSE, *R*^2^, and CNR. The spline technique proposed by Scholkmann et al. (2010) produced the greatest improvement for MSE and *R*^2^, while wavelet analysis described by Molavi and Dumont (2012) produced the greatest increase in CNR. We believe the application of both of these techniques can be highly beneficial to functional NIRS analysis.

## Conflict of Interest Statement

The authors declare that the research was conducted in the absence of any commercial or financial relationships that could be construed as a potential conflict of interest.

## Footnotes

^1^www.nmr.mgh.harvard.edu/optics

^2^www-stat.stanford.edu/~wavelab

^3^www.nmr.mgh.harvard.edu/optics
